# The use of artificial neural networks to study fatty acids in neuropsychiatric disorders

**DOI:** 10.1186/1471-244X-8-S1-S3

**Published:** 2008-04-17

**Authors:** Massimo Cocchi, Lucio Tonello, Sofia Tsaluchidu, Basant K Puri

**Affiliations:** 1 Università di Bologna, Bologna, Italy; 2 MRI Unit, MRC Clinical Sciences Centre, Imaging Sciences Department, Imperial College London, Hammersmith Hospital, Du Cane Road, London W12 0HS, UK

## Abstract

**Background:**

The range of the fatty acids has been largely investigated in the plasma and erythrocytes of patients suffering from neuropsychiatric disorders. In this paper we investigate, for the first time, whether the study of the platelet fatty acids from such patients may be facilitated by means of artificial neural networks.

**Methods:**

Venous blood samples were taken from 84 patients with a DSM-IV-TR diagnosis of major depressive disorder and from 60 normal control subjects without a history of clinical depression. Platelet levels of the following 11 fatty acids were analyzed using one-way analysis of variance: C14:0, C16:0, C16:1, C18:0, C18:1 *n*-9, C18:1 *n*-7, C18:2 *n*-6, C18:3 *n*-3, C20:3 *n*-3, C20:4 *n*-6 and C22:6 *n*-3. The results were then entered into a wide variety of different artificial neural networks.

**Results:**

All the artificial neural networks tested gave essentially the same result. However, one type of artificial neural network, the self-organizing map, gave superior information by allowing the results to be described in a two-dimensional plane with potentially informative border areas. A series of repeated and independent self-organizing map simulations, with the input parameters being changed each time, led to the finding that the best discriminant map was that obtained by inclusion of just three fatty acids.

**Conclusion:**

Our results confirm that artificial neural networks may be used to analyze platelet fatty acids in neuropsychiatric disorder. Furthermore, they show that the self-organizing map, an unsupervised competitive-learning network algorithm which forms a nonlinear projection of a high-dimensional data manifold on a regular, low-dimensional grid, is an optimal type of artificial neural network to use for this task.

## Background

Since phospholipids are the major constituents of neuronal and glial cell membranes, synaptic vesicular membranes, and the membranes of other intracellular organelles such as the nucleus, endoplasmic reticulum, Golgi apparatus and mitochondria, it seems reasonable to study phospholipids and their constituent fatty acids in neuropsychiatric disorders [1,2]. The long-chain polyunsaturated fatty acids which are the constituents of phospholipid molecules are themselves derived from short-chain precursor essential fatty acids via biosynthetic intermediate fatty acids, and may be transformed into other, non-phospholipid fatty acids. Thus, the range of the fatty acids has been largely investigated in the plasma and erythrocytes of patients suffering from neuropsychiatric disorders. In this paper we investigate, for the first time, whether the study of platelet fatty acids from such patients may be facilitated by means of artificial neural networks (ANNs).

We report the first study of the applicability of an artificial neural network to the analysis of platelet fatty acids from a group of patients with a neuropsychiatric disorder.

## Methods

### Fatty acid data

Venous blood samples were taken from 84 patients with a DSM-IV-TR diagnosis of major depressive disorder and from 60 normal control subjects without a history of clinical depression. Platelet levels of the following 11 fatty acids were analyzed using one-way analysis of variance (ANOVA): C14:0, C16:0, C16:1, C18:0, C18:1 *n*-9, C18:1 *n*-7, C18:2 *n*-6, C18:3 *n*-3, C20:3 *n*-3, C20:4 *n*-6 and C22:6 *n*-3.

The study was carried out according to the Declaration of Helsinki. The subjects were given details of the study and gave informed consent. The study was approved by the local research ethics committee.

### ANN

The concentrations of those fatty acids surviving the one-way ANOVA were entered, blind to group status, age or gender, into a wide variety of different types of ANN. These were run on a PC on a C/C++ platform. The results from the different ANNs were compared.

### Statistical analyses

Statistical analyses were carried out using the STATISTICA version 7 data analysis program (StatSoft Inc., Tulsa, OK, USA; ).

## Results

All the ANNs tested gave essentially the same result. However, one type of ANN, known as a self-organizing map (SOM), gave superior information by allowing the results to be described in a two-dimensional plane with potentially informative border areas. A series of repeated and independent SOM simulations, with the input parameters being changed each time, led to the finding that the best discriminant map was that obtained by inclusion of the following three fatty acids: linoleic acid (C18:2 *n*-6), arachidonic acid (C20:4 *n*-6) and palmitic acid (C16:0).

## Discussion

Kohonen [3] has defined an ANN as being a 'massively parallel interconnected network of simple (usually adaptive) elements and their hierarchical organizations, intended to interact with the objects of the real world in the same way as the biological nervous systems do. In a more general sense, artificial neural networks also encompass abstract schemata, such as mathematical estimators and systems of symbolic rules, constructed automatically from masses of examples, without heuristic design or other human intervention. Such schemata are supposed to describe the operation of biological or artificial neural networks in a highly idealized form and define certain performance limits.' The most important types of ANNs are: signal-transfer networks, in which the output signal values depend uniquely on input signals, so that these circuits are designed for signal transformations; state-transfer networks, which are based on relaxation effects, in which the feedbacks and non-linearities are strong enough to cause the activity state rapidly to converge to a stable value (the attractor); and competitive-learning networks, in the simplest structures of which cells compete upon receiving identical input information [3]. The type of ANN we found to be best, namely the self-organizing map (SOM), belongs to the last group.

The SOM is an unsupervised competitive-learning network algorithm, which was invented by Teuvo Kohonen in 1981–82. According to Kohonen et al. [4] 'The central property of the SOM is that it forms a nonlinear projection of a high-dimensional data manifold on a regular, low-dimensional (usually 2D) grid. In the display, the clustering of the data space as well as the metric-topological relations of the data items are clearly visible. If the data items are vectors, the components of which are variables with a definite meaning such as the descriptors of statistical data, or measurements that describe a process, the SOM grid can be used as a groundwork on which each of the variables can be displayed separately using grey-level or pseudocolor coding. This kind of combined display has been found very useful for the understanding of the mutual dependencies between the variables, as well as of the structures of the data set.' In the context of this definition, a manifold refers to a topological space with well-defined mathematical properties. A particular strength of the SOM map displays lies in enabling relevant information to be 'found' rather than 'searched for' [4].

## Conclusion

Our results show that a SOM is an optimal type of ANN to use in the analysis of fatty acids in neuropsychiatric disorders.

## Competing interests

The authors declare that they have no competing interests.

## Authors' contributions

All the authors made substantial contributions to the design and conception of the study. MC and LT were particularly involved in data collection and data analysis. All authors were involved in the interpretation of the data. All the authors have been involved in drafting and revising the manuscript and have read and approved the final manuscript.
